# Genome‐wide association study reveals novel players in defense hormone crosstalk in *Arabidopsis*

**DOI:** 10.1111/pce.13357

**Published:** 2018-07-03

**Authors:** Silvia Proietti, Lotte Caarls, Silvia Coolen, Johan A. Van Pelt, Saskia C.M. Van Wees, Corné M.J. Pieterse

**Affiliations:** ^1^ Plant‐Microbe Interactions, Department of Biology, Science4Life Utrecht University Utrecht The Netherlands

**Keywords:** genome‐wide association (GWA) mapping, hormone crosstalk, *Botrytis cinerea*, *Mamestra brassicae*, salicylic acid, abscisic acid, jasmonic acid

## Abstract

Jasmonic acid (JA) regulates plant defenses against necrotrophic pathogens and insect herbivores. Salicylic acid (SA) and abscisic acid (ABA) can antagonize JA‐regulated defenses, thereby modulating pathogen or insect resistance. We performed a genome‐wide association (GWA) study on natural genetic variation in *Arabidopsis thaliana* for the effect of SA and ABA on the JA pathway. We treated 349 *Arabidopsis* accessions with methyl JA (MeJA), or a combination of MeJA and either SA or ABA, after which expression of the JA‐responsive marker gene *PLANT DEFENSIN1.2* (*PDF1.2*) was quantified as a readout for GWA analysis. Both hormones antagonized MeJA‐induced *PDF1.2* in the majority of the accessions but with a large variation in magnitude. GWA mapping of the SA‐ and ABA‐affected *PDF1.2* expression data revealed loci associated with crosstalk. *GLYI4* (encoding a glyoxalase) and *ARR11* (encoding an *Arabidopsis* response regulator involved in cytokinin signalling) were confirmed by T‐DNA insertion mutant analysis to affect SA–JA crosstalk and resistance against the necrotroph *Botrytis cinerea*. In addition, *At1g16310* (encoding a cation efflux family protein) was confirmed to affect ABA–JA crosstalk and susceptibility to *Mamestra brassicae* herbivory. Collectively, this GWA study identified novel players in JA hormone crosstalk with potential roles in the regulation of pathogen or insect resistance.

## Abstract 2

Crosstalk between defense‐related hormones enables plants to finely tune their immune response to the plethora of attackers that they encounter in their environment. We mined the natural genetic variation in *Arabidopsis thaliana* for novel genes that are associated with positive or negative interactions that the stress hormones salicylic acid and abscisic acid have on jasmonic acid‐regulated defense responses. Through genome‐wide association mapping, we identified three novel genes with putative roles in hormone crosstalk and that impact the level of resistance against the necrotrophic pathogen *Botrytis cinerea* or the insect herbivore *Mamestra brassicae*.

## 1 INTRODUCTION

Plants are continuously attacked by harmful microbial pathogens and herbivorous insects. To defend themselves against these diverse stresses, plants have evolved highly regulated defense systems, largely orchestrated by small‐molecule hormones, such as salicylic acid (SA), jasmonic acid (JA), ethylene (ET), and abscisic acid (ABA; Pieterse, Van der Does, Zamioudis, Leon‐Reyes, & Van Wees, 2012, Robert‐Seilaniantz, Grant, & Jones, 2011, Vos, Pieterse, & Van Wees, 2013, Vos, Verhage et al., 2013). SA generally induces plant defenses against biotrophic pathogens (Glazebrook, 2005). JA and ET are important hormonal regulators of induced plant defenses against necrotrophic pathogens, whereas JA works in concerted action with ABA to induce plant defenses against herbivorous insects (Pieterse et al., 2012). To respond effectively to each attacker or to multiple attackers at the same time, hormonal signalling pathways cross communicate in antagonistic or synergistic manners. In particular, SA and ABA have been shown to interact with the JA pathway, thereby strongly modulating the JA‐induced defense output (Pieterse et al., 2012).

In *Arabidopsis thaliana* (hereafter *Arabidopsis*), two distinct branches of the JA pathway have been shown to antagonize each other: the ethylene response factor (ERF) branch and the MYC branch, which are coregulated by ET and ABA, respectively (Hickman et al., 2017; Pieterse et al., 2012; Robert‐Seilaniantz et al., 2011). The ERF branch of the JA pathway is typically activated upon infection by necrotrophic pathogens and is regulated by the APETALA2 (AP2)/ERF)‐domain transcription factors ERF1 and ORA59 (for OCTADECANOID‐RESPONSIVE ARABIDOPSIS AP2/ERF domain protein 59; Anderson et al., 2004; Lorenzo, Piqueras, Sánchez‐Serrano, & Solano, 2003; Pré et al., 2008). Induction of the ERF branch results in the activation of a large set of JA/ET‐responsive genes, including the marker gene *PLANT DEFENSIN1.2* (*PDF1.2*; Lorenzo et al., 2003, Penninckx, Thomma, Buchala, Métraux, & Broekaert, 1998). The MYC branch of the JA pathway is typically activated upon wounding or feeding by herbivorous insects and is regulated by the basic helix–loop‐helix leucine zipper transcription factors MYC2, MYC3, and MYC4 (Anderson et al., 2004; Fernandez‐Calvo et al., 2011; Niu, Figueroa, & Browse, 2011; Vos, Verhage et al., 2013). Activation of the MYC branch leads to transcription of a large set of JA‐responsive genes, including the marker genes *VEGETATIVE STORAGE PROTEIN1* (*VSP1*) and *VSP2* (Anderson et al., 2004; Lorenzo et al., 2004).

SA has been reported to have a major impact on JA‐induced defenses in both the ERF and the MYC branch of the JA pathway (Bostock, 2005; Pieterse et al., 2012; Stout, Thaler, & Thomma, 2006). Although the effect of SA on the JA pathway can be antagonistic, synergistic, or neutral, in *Arabidopsis*, antagonistic interactions seem to prevail (Pieterse et al., 2012; Tsuda, Sato, Stoddard, Glazebrook, & Katagiri, 2009). Experiments performed with *Arabidopsis* revealed that the JA‐responsive genes *PDF1.2* and *VSP2* are highly sensitive to suppression by SA. In many cases, this antagonism between the SA and JA pathways affects plant resistance against necrotrophs or insect herbivores (Caarls, Pieterse, & Wees, 2015). Suppression of the JA pathway by SA is predominantly regulated at the level of gene transcription (Caarls et al., 2015; Van der Does et al., 2013). Important regulators of the interaction between the SA and JA pathways have been identified, such as the redox sensitive transcriptional coregulator NONEXPRESSOR OF PATHOGENESIS‐RELATED PROTEINS1 (NPR1; Spoel et al., 2003) and several WRKY and TGA transcription factors (Caarls et al., 2015). SA‐induced redox changes mediated by thioredoxins and glutaredoxins modify the activity of transcriptional regulators that are involved in suppression of JA‐dependent genes, such as NPR1 and TGAs (Ndamukong et al., 2007; Tada et al., 2008; Zander, Chen, Imkampe, Thurow, & Gatz, 2012). SA‐induced negative regulators of JA‐responsive gene expression have been identified as well, including the WRKY transcription factors WRKY50, WRKY51, and WRKY70 (Gao, Venugopal, Navarre, & Kachroo, 2011), while a role for SA‐responsive ERF‐type transcriptional repressors was ruled out (Caarls et al., 2017). Moreover, SA was shown to promote degradation of the transcription factor *ORA59* (Van der Does et al., 2013) and to inhibit *ORA59* gene expression (Zander, Thurow, & Gatz, 2014), providing a mechanistic explanation of how SA suppresses the ERF branch of the JA pathway.

Like SA, ABA is also a strong modulator of JA‐induced defenses. When produced in combination with JA, ABA acts synergistically on the expression of the MYC branch of the JA pathway while it antagonizes the ERF branch and, thus, suppresses JA‐induced *PDF1.2* expression (Abe et al., 2003; Anderson et al., 2004; Pieterse et al., 2012; Verhage et al., 2011; Vos, Moritz, Pieterse, & Van Wees, 2015). This results in prioritization of the immune signalling network toward the MYC branch of the JA pathway, which is associated with resistance to herbivory (Anderson et al., 2004; Bodenhausen & Reymond, 2007; Dombrecht et al., 2007; Fernandez‐Calvo et al., 2011), while resistance to necrotrophs is compromised (Anderson et al., 2004). For example, in *MYC2*‐mutated *jin1* and ABA biosynthesis mutant *aba2‐1* plants, the ERF branch of the JA pathway is no longer inhibited, resulting in increased *PDF1.2* expression and enhanced resistance against necrotrophic pathogens, such as *Botrytis cinerea, Plectosphaerella cucumerina*, and *Fusarium oxysporum* (Adie, Chico, Rubio‐Somoza, & Solano, 2007; Anderson et al., 2004; Lorenzo et al., 2004; Nickstadt et al., 2004; Sánchez‐Vallet et al., 2012). Furthermore, caterpillars of the insect herbivore *Pieris rapae* preferred to feed from mutant *jin1* plants and *ORA59*‐overexpressing plants over wild‐type plants (Verhage et al., 2011; Vos et al., 2015), indicating that crosstalk between the ERF and the MYC branch also affects plant–insect interactions. Beside the mentioned MYC transcription factors, transcription factors of the R2R3‐MYB, NAC, and WRKY family have also been identified as potential hubs in ABA–JA crosstalk (AbuQamar, Luo, Laluk, Mickelbart, & Mengiste, 2009; Chen et al., 2010; Dombrecht et al., 2007; Nakashima et al., 2007).

Using a small set of 18 *Arabidopsis* accessions, we previously demonstrated that all tested accessions were sensitive to SA‐mediated suppression of the JA‐responsive marker gene *PDF1.2*, albeit to different extents (Koornneef et al., 2008), highlighting the potential significance of crosstalk between the SA and JA pathway in induced plant defenses in nature (Thaler, Humphrey, & Whiteman, 2012). We reasoned that the observed natural genetic variation in the level of hormone crosstalk would provide a so far unexplored resource from which genes encoding novel players in hormone crosstalk could be identified. Recent advances in genotyping and sequencing technology have made genome‐wide association (GWA) mapping a good approach to mine natural genetic variation and detect molecular markers linked to, for example, stress resistance traits (Assmann, 2013; Atwell et al., 2010; Meijon, Satbhai, Tsuchimatsu, & Busch, 2014). GWA mapping is a method initially utilized in human population studies to identify the genetic basis of complex traits (Hirschhorn & Daly, 2005). GWA mapping has also been successfully utilized in plant studies (Aranzana et al., 2005; Atwell et al., 2010; Bac‐Molenaar et al., 2015; Broekgaarden et al., 2015; Chan, Rowe, & Kliebenstein, 2010; Kloth, Thoen, Bouwmeester, Jongsma, & Dicke, 2012; Li, Huang, Bergelson, Nordborg, & Borevitz, 2010; Thoen et al., 2017; Wintermans, Bakker, & Pieterse, 2016). The underlying rationale of GWA mapping is that natural variation in the phenotype of a given trait is caused by genetic differences in the population under study. Via single nucleotide polymorphisms (SNPs) in the genomes of the phenotyped genotypes, these genetic differences can be linked to genetic loci and ideally to the responsible genes. In this study, we used the GWA mapping resource of the *Arabidopsis* Haplotype Map (HapMap) collection that is comprised of over 360 natural accessions collected globally and genotyped for ~250k SNPs relative to the Col‐0 accession (Clark et al., 2007; Kim et al., 2007; Nordborg et al., 2005; Weigel & Mott, 2009). We observed a large genetic variation in the magnitude by which SA and ABA affect the expression of the JA‐responsive marker gene *PDF1.2* in the different *Arabidopsis* accessions. GWA mapping revealed multiple loci associated with either SA–JA or ABA–JA crosstalk. Several candidate genes were validated using T‐DNA insertion mutant analysis, which yielded a number of novel players in SA–JA and ABA–JA crosstalk with effects on the level of resistance against the necrotrophic pathogen *B. cinerea* or the herbivorous insect *Mamestra brassicae*.

## 2 RESULTS

### 2.1 Natural genetic variation in *Arabidopsis* for the effect of SA and ABA on MeJA‐induced *PDF1.2* expression

**Figure 1:**
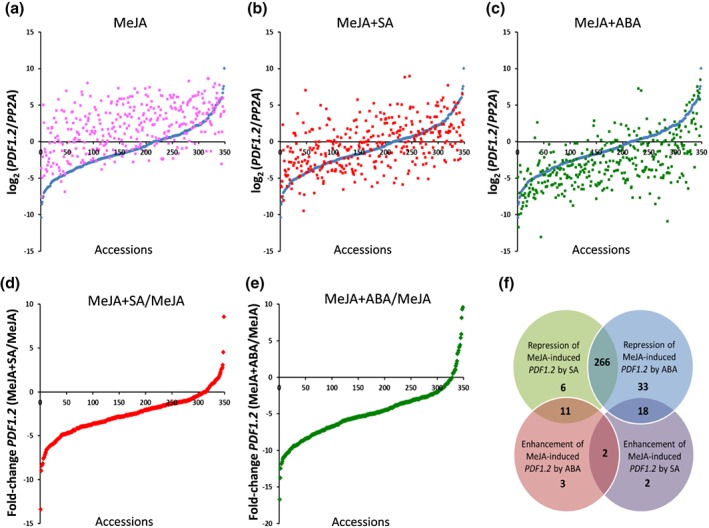
Natural variation in 349 wild *Arabidopsis* accessions for *PLANT DEFENSIN1.2* (*PDF1.2*) transcript levels after treatment with methyl JA (MeJA) or a combination of MeJA and either salicylic acid (SA) or abscisic acid (ABA)**.** (a) *PDF1.2* transcript levels relative to those of the constitutively expressed *Arabidopsis* reference gene *PP2AA3* (At1g13320) in leaves of control (blue dots) or MeJA‐treated plants (pink dots). (b) *PDF1.2* transcript levels in leaves of control (blue dots) or a combination of MeJA and SA (red dots). (c) *PDF1.2* transcript levels in leaves of control (blue dots), or a combination of MeJA and ABA (green dots). (d) Fold change in *PDF1.2* expression in MeJA + SA‐ over MeJA‐treated plants. (e) Fold change in *PDF1.2* expression in MeJA + ABA‐ over MeJA‐treated plants. For each accession, gene expression analyses were performed 24 hr after hormone treatment of 4‐week‐old plants. In panels a–c, accessions are similarly sorted on the level of *PDF1.2* expression in the respective control plants (blue dots). In panels d–e, accessions are sorted on the magnitude of the effect of the respective hormone treatments. Hence, the order of the accessions differs in these panels. (f) The Venn diagram shows the overlap between the accessions that display repression (green) or enhancement (purple) of MeJA‐induced *PDF1.2* by SA and repression (blue) or enhancement (pink) of MeJA‐induced *PDF1.2* by ABA. Only accessions showing >2‐fold change in *PDF1.2* expression in the combination treatment with MeJA over the treatment with MeJA alone are included in the comparison (305 accessions for MeJA + SA and 333 accessions for MeJA + ABA)

To analyse the natural genetic variation in *Arabidopsis* for the effect of SA and ABA on JA‐responsive gene expression, we quantified the level of *PDF1.2* transcription in 349 *Arabidopsis* accessions, 24 hr after treatment of the leaves with MeJA, or a combination of MeJA and SA or ABA (Figure 1; Table S1). Figure 1a shows that the basal level of *PDF1.2* expression varied among accessions (Figure 1a, blue dots) and that most of the accessions (305 out of 349) showed induced levels of *PDF1.2* expression 24 hr after application of MeJA, albeit with different magnitudes (Figure 1a, pink dots). In the combination treatment with SA, 283 accessions displayed a >2‐fold lower level of *PDF1.2* expression than in the MeJA treatment alone (Figure 1a and 1d; Table S1). Similarly, in the MeJA + ABA combination treatment, 317 accessions showed a >2‐fold lower level of *PDF1.2* expression than in the MeJA treatment alone (Figure 1c and 1e; Table S1). Again, the collection of *Arabidopsis* accessions displayed large natural variation in the magnitude by which MeJA‐induced *PDF1.2* was affected by SA and ABA (Figure 1d and 1e). In only a small number of accessions, SA and ABA enhanced the level of MeJA‐induced *PDF1.2* by >2‐fold (22 and 16 accessions, respectively). Together, these results indicate that *Arabidopsis* possesses a large natural genetic variation in the magnitude by which SA and ABA affect JA‐responsive gene expression.

To gain insight in the correlation between the effect of SA and ABA on MeJA‐induced *PDF1.2* expression, we first grouped the accessions in terms of their response to SA and ABA, respectively. Figure 1f shows that a majority of 266 accessions displayed a >2‐fold repression of MeJA‐induced *PDF1.2* in response to both SA and ABA. Only two accessions showed enhanced MeJA‐induced *PDF1.2* expression in response to either of these hormones. To test whether the magnitude and direction of the effect of SA in the different accessions is correlated with that observed in response to ABA, we performed a Spearman correlation test on the fold‐change *PDF1.2* expression values in the MeJA + SA and the MeJA+ABA combination treatments relative to those of the MeJA single treatment. The test yielded only a moderate correlation (r = 0.40), suggesting that the effects of SA and ABA on MeJA‐induced *PDF1.2* are not strongly linked.

### 2.2 GWA mapping reveals loci associated with the effect of SA and ABA on JA‐responsive gene expression

**Figure 2:**
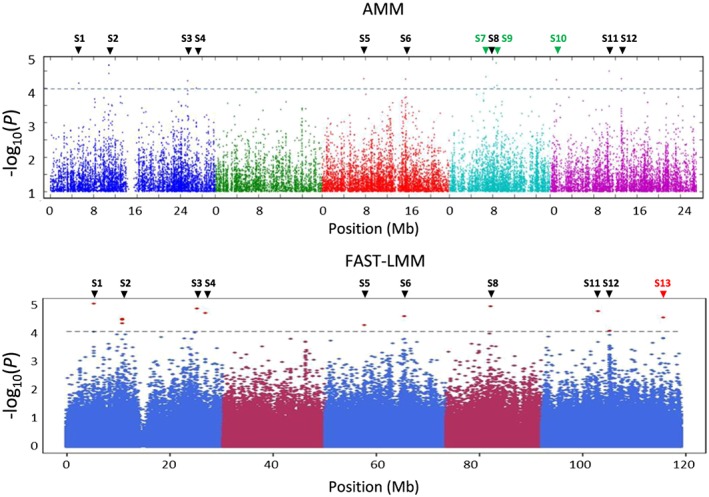
Genome‐wide association mapping results of salicylic acid‐mediated effect on methyl JA‐induced *PLANT DEFENSIN1.2* expression. Manhattan plot of the –log_10_(*p*) single nucleotide polymorphism marker‐trait associations performed by accelerated mixed model (AMM) and FAST‐LMM. From left to right, different colors represent *Arabidopsis* chromosomes I‐V. The dotted grey line indicates the arbitrary threshold of –log_10_(*p*) = 4. *Arrows* indicate loci with significant single nucleotide polymorphism‐trait associations in the AMM *(*green), FAST‐LMM (red), or both AMM and FAST‐LMM analysis (black)

**Figure 3:**
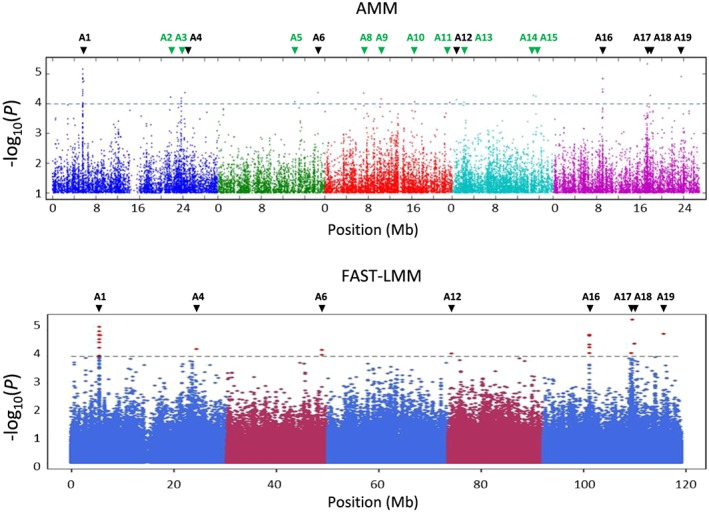
Genome‐wide association mapping results of abscisic acid‐mediated effect on methyl JA‐induced *PLANT DEFENSIN1.2* expression. Manhattan plot of the –log_10_(*p*) single nucleotide polymorphism marker‐trait associations performed by accelerated mixed model (AMM) and FAST‐LMM. From left to right, different colors represent *Arabidopsis* chromosomes I‐V. The dotted grey line indicates the arbitrary threshold of –log_10_(*p*) = 4. Arrows indicate loci with significant SNP‐trait associations in the AMM (green) or both AMM and FAST‐LMM analysis (black)

To identify novel players involved in the effect of SA and ABA on JA‐responsive gene expression, we performed GWA mapping using the ~214k SNP set that is commonly used for GWA studies in *Arabidopsis* (Atwell et al., 2010; Bac‐Molenaar et al., 2015; Horton et al., 2012; Kim et al., 2007; Li, et al., 2010). To this end, we used normally distributed log_2_ *PDF1.2* gene expression data of the MeJA + SA treatment and the MeJA+ABA treatment, relative to *PDF1.2* expression data of the single MeJA treatment [log_2_ (*PDF1.2* ^*MeJA+SA*^ */PDF1.2* ^*MeJA*^)] and [log_2_ (*PDF1.2* ^*MeJA+ABA*^ */PDF1.2* ^*MeJA*^)], respectively (Table S1). Out of 349 accessions, SNP data for 335 accessions were available in the GWAPP tool that we used for GWA mapping (Seren et al., 2012). Of these, data from 327 accessions of the MeJA + SA/MeJA dataset and 322 accessions of the MeJA + ABA/MeJA dataset were normally distributed (Kolmogorov–Smirnov test, *p* = 0.05) and thus included in the GWA analysis. Subsequently, GWA mapping was carried out on the MeJA + SA/MeJA and the MeJA + ABA/MeJA datasets using the accelerated mixed model (AMM) and the FAST–LMM algorithm (Cao et al., 2011; Seren et al., 2012). A minor allele frequency (MAF) >5% and a –log_10_(*p*) > 4 was used as a threshold to detect SNP‐trait associations. Manhattan plots from AMM and FAST–LMM show several peaks with single or multiple SNP‐trait associations (Figure 2 and Figure 3). To reduce false positives and negatives, we selected only SNP‐trait associations that were found to be significant by both AMM and FAST‐LMM. For the MeJA + SA/MeJA dataset, 10 SNPs, representing nine loci were found to be significantly associated with SA‐mediated suppression of MeJA‐induced *PDF1.2* expression. For the MeJA + ABA/MeJA dataset, we identified 25 SNPs, representing eight loci to be significantly associated with ABA‐mediated suppression of MeJA‐induced *PDF1.2* expression. There is no overlap in the two sets of identified loci, confirming that the effects of SA and ABA on MeJA‐induced *PDF1.2* are not likely to be linked. In *Arabidopsis,* the linkage disequilibrium of the population of *Arabidopsis* accessions used for GWA mapping is estimated 10–50 kb (Kim et al., 2007; Nordborg et al., 2005). Therefore, we considered all genes within 15 kb upstream and downstream of each significant SNP to be candidates for the observed SNP‐trait associations. This yielded a list of 60 candidate genes in nine loci for the MeJA + SA/MeJA dataset and 98 candidate genes in eight loci for the MeJA + ABA/MeJA dataset (Table S2). Their putative functions based on the Gene Ontology tool of The *Arabidopsis* Information Resource (Lamesch et al., 2012) are given in Table S2.

### 2.3 Fine mapping of GWA‐identified loci

To validate the identified SNP‐trait associations in the GWA mapping analyses, we performed locus‐specific mapping (LSM) using whole genome sequences of *Arabidopsis* accessions that were downloaded from the SALK 1001 genomes project. Alignment of complete genome sequences from different accessions provides all polymorphic markers compared with the Col‐0 accession‐based SNP data present in the GWA tools, and hence increases the resolution of the mapping. Among the 327 accessions of the MeJA + SA/MeJA dataset used for GWA mapping, genomes of 153 accessions were available in the SALK 1001 genomes database. For the MeJA + ABA/MeJA dataset, genomes of 147 of the 322 accessions were available. LSM was performed on the MeJA + SA/MeJA and MeJA + ABA/MeJA data of the selected accessions (153 and 147 accessions, respectively; accessions used (1) or not (0) in LSM analysis are indicated in Table S1). In the 15 kb upstream and downstream of each significant SNP identified by both AMM and FAST–LMM (–log_10_(*p*) > 4; MAF > 5%), SNP‐trait associations were identified by LSM. The significance of associations between traits and SNP markers was evaluated using a nonparametric Kruskal–Wallis test (Filiault & Maloof, 2012). For the MeJA + SA/MeJA dataset, LSM identified SNP‐trait associations in six of the nine loci that were revealed by GWA mapping (Kruskal–Wallis test; *p*>0.05). Comparing the candidate genes in the genomic regions 15 kb upstream or downstream of each SNP revealed an overlap of eight candidate genes in the loci identified by GWA mapping and LSM (Table 1). For the MeJA + ABA/MeJA data set, LSM identified significant SNP‐trait associations in six of the eight loci that were revealed by GWA mapping. Comparing the candidate genes in the genomic regions 15 kb upstream or downstream of each SNP revealed an overlap of 28 candidate genes in the loci identified by GWA mapping and LSM (Table 2). We considered the candidate genes identified by both GWA and LSM to have the highest probability of being truly associated with the phenotyped traits, hence, we proceeded with these genes for further study. Of the eight selected candidate genes associated with SA–JA crosstalk, we were able to obtain homozygous T‐DNA insertion mutants for six genes (Table 1), whereas of the 28 candidate genes associated with ABA–JA crosstalk, we obtained homozygous T‐DNA insertion mutants for 12 genes (Table 2). The selected candidate genes play a role in a broad range of biological processes, ranging from signal transduction to macromolecules biosynthesis.

**Table 1:** List of candidate genes associated with SA‐mediated effects on MeJA‐induced *PDF1.2* expression as revealed by both GWA mapping and LSM

Candidate gene (AGI)	Gene annotation	GO biological processes	Homozygous T‐DNA insertion lines	‐log_10_(*p*) [AMM]	‐log_10_(*p*) [FAST‐LMM]	‐log_10_(*p*) [LSM]
At1g15380	GLYI4, Lactoglutathione lyase/glyoxylase I family protein	Carbohydrate metabolic process	Yes	4.1	5.0	4.2
At1g15410	Aspartate‐glutamate racemase family	Cellular amino acid metabolic process	Yes	4.1	5.0	4.2
At1g30510	RFNR2, root FNR2	Oxidation–reduction process, photosynthesis	Yes	4.6	4.4	4.3
At1g30550	S‐adenosyl‐L‐methyonine‐dependent methyltrans‐ferases superfamily protein	7‐methylguanosine RNA capping, RNA methylation	Yes	4.6	4.4	4.5
At1g67710	ARR11, Arabidopsis response regulator 11	Cytokinin‐activated signalling pathway, phosphorelay signal transduction system	Yes	4.2	4.8	4.7
At1g71460	Pentatricopeptide repeat (PPR‐like) superfamily protein	Unknown	Yes	4.0	4.6	5.2
At3g21770	Peroxidase superfamily protein	Hydrogen peroxide catabolic process, oxidation‐reduction process	No	4.2	4.2	4.0
At4g15130	CCT2, Phosphorylcholine cytidylyltransferase2	Phosphatidylcholine `biosynthetic process	No	4.7	4.9	4.5

*Note*. Shown are Arabidopsis gene identifier (AGI) numbers of candidate genes that are located in the closest proximity of the identified highly associated SNPs [log_10_(*p*)>4] identified by AMM, FAST‐LMM, and LSM. Their annotation and ontology in TAIR10 and the availability of homozygous T‐DNA insertion lines are also provided. AMM: accelerated mixed model; GWA: genome‐wide association; JA: jasmonic acid; LSM: locus‐specific mapping; MeJA: methyl JA; *PDF1.2*: *PLANT DEFENSIN1.2*; SA: salicylic acid; SNP: single nucleotide polymorphismc; TAIR: The *Arabidopsis* Information Resource.

**Table 2:** List of candidate genes associated with ABA‐mediated effects on MeJA‐induced *PDF1.2* expression as revealed by both GWA mapping and LSM

Candidate gene (AGI)	Gene annotation	GO biological processes	Homozygous T‐DNA insertion lines	‐log_10_(*p*) [AMM]	‐log_1_0(*p*) [FAST‐LMM]	‐log_10_(*p*) [LSM]
At1g16225	Target SNARE coiled‐coil domain protein	No annotation	No	4.3	4.0	4.7
At1g16230	Target SNARE coiled‐coil domain protein	No annotation	No	4.3	4.0	4.9
At1g16240	ATSYP51; synthaxin of plant 51	Intracellular protein transport, vesicle docking	Yes	4.3	4.0	4.3
At1g16260	Wall‐associated kinase family protein	Protein phosphorylation	Yes	4.5	4.5	4.2
At1g16270	Protein kinase superfamily protein with octicosapeptide/Phox/Bem1p domain	Protein phosphorylation	Yes	4.5	4.5	5.0
At1g16310	Cation efflux family protein	Cation transport	Yes	4.5	4.5	4.2
At1g16320	Uncharacterized conserved protein (DUF2358)	No annotation	No	4.8	4.8	4.7
At1g16330	CYCB3;1, CYCLIN B3;1	Regulation of cell cycle	No	4.8	4.8	4.7
At1g16340	ATKDO8PS, ATKDSA2	Biosynthetic process, keto‐3‐deoxy‐D‐manno‐octulosonic acid biosynthetic process	No	4.8	4.8	4.5
At1g16350	Aldolase‐type TIM barrel family protein	GMP biosynthetic process, oxidation‐reduction process	No	5.2	5.1	4.5
At1g16360	LEM3 (ligand‐effect modulator 3) family protein/CDC50 family protein	No annotation	Yes	5.2	5.1	4.3
At1g16370	ATOCT6, Organic Cation/Carnitine Transporter 6	Cellular response to salt, ion transport	Yes	4.8	4.8	5.0
At1g16380	ATCHX1, Cation Exchanger 1	Cation transport, potassium ion transport	No	4.4	4.3	5.1
At1g16390	ATOCT3, Organic cation/Carnitine transporter 3	Cellular response to cold, ion transport	No	4.4	4.3	4.5
At1g16400	Cytochrome P450, family 79, subfamily F, polypeptide 2", CYP79F2	Defense response to other organism, glucosinolate biosynthetic process	No	4.3	4.3	4.6
At1g16410	CYP79F1, Cytochrome P450 79F1	Defense response to other organism, glucosinolate biosynthetic process	Yes	4.3	4.3	4.0
At1g16420	*Arabidopsis thaliana* metacaspase 8, ATMC8	Hydrogen peroxide‐mediated programmed cell death	No	4.3	4.3	4.0
At1g16445	S‐adenosyl‐L‐methionine‐dependent methyltransferases superfamily protein	No annotation	No	4.3	4.3	4.5
At1g65610	*Arabidopsis thaliana* glycosyl hydrolase 9A2, ATGH9A2, ATKOR2	Cell wall organization, cellulose catabolic process	Yes	4.4	4.3	4.2
At1g65630	DEG3, DEGP protease 3, Degradation of periplasmic proteins 3	Proteolysis	No	4.4	4.3	6.1
At4g01820	ABCB3, ATP‐Binding cassette B3, MDR3, P‐Glycoprotein 3, PGP3	Basipetal auxin transport, transmembrane transport	No	4.1	4.1	4.0
At4g01850	AtSAM2, MAT2, S‐adenosylmethionine synthetase 2	S‐adenosylmethionine biosynthetic process, cellular response to iron	Yes	4.1	4.1	4.2
At4g01860	Transducin family protein/WD‐40 repeat family protein	No annotation	Yes	4.1	4.1	4.1
At4g01880	Methyltransferases	No annotation	No	4.1	4.1	4.5
At5g43210	Excinuclease ABC, C subunit, N‐terminal	DNA repair	No	5.3	5.4	4.9
At5g44380	ATBBE28	Oxidation–reduction process, response to oxidative stress	No	4.3	4.5	4.1
At5g58410	HEAT/U‐box domain‐containing protein	Protein ubiquitination	Yes	4.9	4.8	4.6
At5g58412	Plant thionin family protein	No annotation	Yes	4.9	4.8	4.1

Shown are Arabidopsis AGI numbers of candidate genes that are located in the closest proximity of the identified highly‐associated SNPs [log_10_(*p*)>4] identified by AMM, FAST‐LMM, and LSM. Their annotation and ontology in TAIR10, and the availability of homozygous T‐DNA insertion lines are also provided. *Note*. ABA: abscisic acid; AMM: accelerated mixed model; GWA: genome‐wide association; JA: jasmonic acid; LSM: locus‐specific mapping; MeJA: methyl JA; *PDF1.2*: *PLANT DEFENSIN1.2*; SNP: single nucleotide polymorphismc; TAIR: The *Arabidopsis* Information Resource.

### 2.4 T‐DNA insertion line analysis of candidate genes associated with SA–JA crosstalk

**Figure 4:**
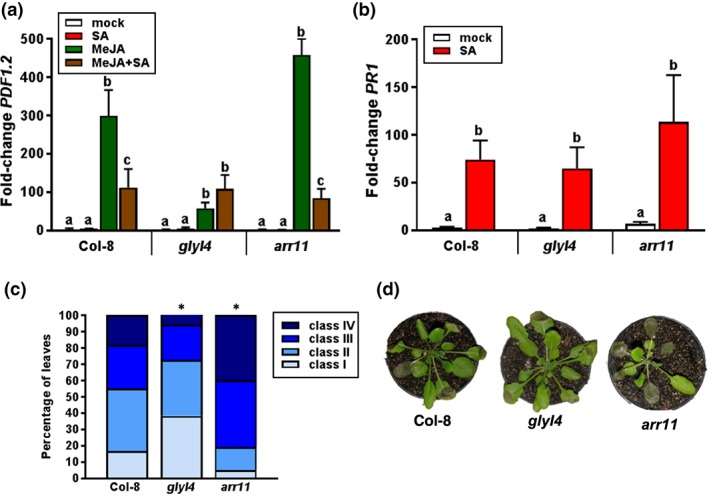
Validation of candidate genes associated with salicylic acid (SA)–jasmonic acid (JA) crosstalk and resistance against *Botrytis cinerea*. (a–b) qRT‐PCR analysis of (a) *PLANT DEFENSIN1.2* and (b) *PR1* transcript levels in leaves of Col‐8 and T‐DNA insertion mutants *glyI4* and *arr11* that were treated with SA, methyl JA (MeJA), or MeJA + SA. Fold change in gene expression levels are calculated relative to mock‐treated Col‐8 plants and normalized to the reference gene *PP2AA3* (At1g13320). Gene expression analyses were performed 24 hr after hormone treatment of 5‐week‐old plants. Shown data are means of three biological replicates. Error bars represent standard error of the mean (SEM). Different letters represent statistically significant differences between treatments (two‐way analysis of variance, Tukey's test; *p* < 0.05). (c) Distribution of disease symptoms of leaves of Col‐8, *glyI4*, and *arr11*, 3 days after inoculation with *B. cinerea*. Disease severity of inoculated leaves was scored in four classes ranging from restricted lesion (Class I), nonspreading lesion (Class II), spreading lesion (Class III), up to severely spreading lesion (Class IV). The percentage of leaves in each class was calculated per plant (*n* = 24). Asterisk indicates statistically significant difference from Col‐8 (χ^2^ test; *p <* 0.05). (d) Photographs of *B. cinerea* disease symptoms on Col‐8, *glyI4,* and *arr11*, 3 days after inoculation. The experiments have been repeated with similar results

To investigate whether the selected candidate genes (Table 1) have a role in SA–JA crosstalk, we tested the effect of SA on MeJA‐induced *PDF1.2* expression in wild‐type accession Col‐8 and in homozygous T‐DNA insertion lines of the respective candidate genes (Table S3). *PDF1.2* expression was monitored in 5‐week‐old plants, 24 hr after exogenous application of MeJA or a combination of MeJA + SA. *PDF1.2* expression levels were compared with the *PDF1.2* expression level in MeJA‐treated Col‐8. While T‐DNA insertion mutant lines for genes *At1g15410, At1g30510*, *At1g30550,* and *At1g71460* did not show consistent altered phenotypes in comparison to Col‐8 (Figure S1), T‐DNA insertion mutants *glyI4* and *arr11* performed consistently different from wild‐type Col‐8 over experiments. In Col‐8, MeJA‐induced *PDF1.2* expression was significantly suppressed by SA (Figure 4a). However, in mutant *glyI4*, SA had no effect on the level of MeJA‐induced *PDF1.2* transcription. Conversely, mutant *arr11* showed a stronger SA‐mediated suppression of MeJA‐induced *PDF1.2* than did Col‐8. To test if this is due to an altered SA sensitivity of the mutants, we tested the responsiveness of *glyI4* and *arr11* to SA by determining the expression level of the SA‐responsive marker gene *PR‐1* in response to exogenous application of 1 mM of SA. Figure 4b shows that *PR‐1* transcript levels accumulated to similar levels in SA‐treated Col‐8, *glyI4*, and *arr11* plants. Hence, the altered SA–JA crosstalk phenotypes of *glyI4* and *arr11* are not likely to be caused by changes in SA sensitivity.

In *Arabidopsis*, *B. cinerea* induces JA‐dependent defenses in the plant that effectively suppress disease (Thomma et al., 1998, Windram et al., 2012). In turn, *B. cinerea* has been shown hijack the SA pathway to suppress effective JA‐dependent defenses via SA–JA crosstalk (El Oirdi et al., 2011). Therefore, we next tested *glyI4* and *arr11* for their level of resistance against *B. cinerea*. To this end, 5‐week‐old plants were inoculated with *B. cinerea* spores, and disease symptoms were scored 3 days later. Figure 4c and 4d show that *glyI4* developed significantly less‐severe disease symptoms than Col‐8. Conversely, *arr11* developed significantly more‐severe disease symptoms than Col‐8. Together, these results indicate that reduced SA–JA crosstalk in *glyI4* and enhanced SA–JA crosstalk in *arr11,* contrastingly affect the level of resistance against *B. cinerea*. Loss of SA–JA crosstalk correlates with enhanced resistance, while increased SA–JA crosstalk is associated with enhanced susceptibility to this necrotrophic pathogen.

### 2.5 T‐DNA insertion lines analysis of candidate genes associated with ABA–JA crosstalk

**Figure 5:**
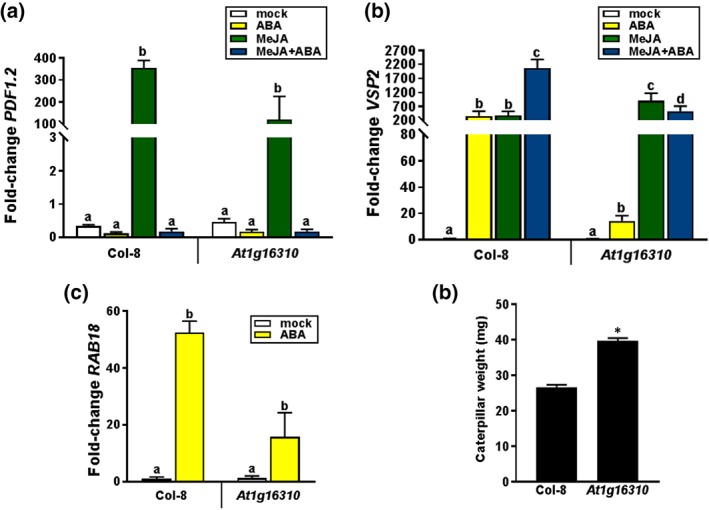
Validation of candidate genes associated with abscisic acid (ABA)–jasmonic acid (JA) crosstalk and resistance against *Mamestra brassicae*. (a–c) qRT‐PCR analysis of (a) *PLANT DEFENSIN1.2* (*PDF1.2*), (b) *VEGETATIVE STORAGE PROTEIN2* (*VSP2*), and (c) *RAB18* transcript levels in leaves of Col‐8 and T‐DNA insertion mutant *At1g16310* that were treated with ABA, methyl JA (MeJA), or MeJA + ABA. Fold change in gene expression levels are calculated relative to mock‐treated Col‐8 plants and normalized to the reference gene *PP2AA3* (At1g13320). Gene expression analyses were performed 5 hr (*VSP2* and *RAB18*) or 24 hr (*PDF1.2*) after hormone treatment. Shown data are means of three biological replicates. Error bars represent SEM. Different letters represent statistically significant differences between treatments (two‐way ANOVA, Tukey's test; *p* < 0.05). (d) *M. brassicae* caterpillar performance on Col‐8 and T‐DNA insertion mutant *At1g16310*. One first‐instar larva of *M. brassicae* was placed on each plant and allowed to feed for 14 days after which the weight of the caterpillar was determined. Asterisk indicates a statistically significant difference in comparison to Col‐8 (Tukey post hoc test; *p* < 0.05). Error bars represent SEM (*n* = 15−24). The experiments have been repeated with similar results

To investigate whether the selected candidate genes (Table 2) have a role in ABA–JA crosstalk, we tested the effect of ABA on MeJA‐induced *PDF1.2* expression in Col‐8 and in homozygous T‐DNA insertion lines of the respective candidate genes (Table S3). *PDF1.2* expression was analysed in 5‐week‐old plants 24 hr after exogenous application of MeJA or a combination of MeJA + ABA. *PDF1.2* expression levels were compared with the *PDF1.2* expression level in MeJA‐treated Col‐8. While T‐DNA insertion mutant lines for genes *At1g16240, At1g16260, At1g16270, At1g16360, At1g16410, At1g16370, At1g65610, At4g01850, At4g01860, At5g58410,* and *At5g58412* did not show consistent altered phenotypes in comparison with Col‐8 (Figure S2), the mutant with a T‐DNA insertion in *At1g16310* (encoding an uncharacterized cation efflux family protein) displayed a four‐fold weaker level of ABA‐mediated suppression of MeJA‐induced *PDF1.2* expression than did Col‐8 (Figure 5), which was consistent over experiments. This weaker ABA–JA crosstalk seemed to be mainly because of the fact that the level of induction by MeJA was reduced in mutant *At1g16310*. To further investigate this, we also tested the expression of *VSP2* in response to treatment with MeJA, ABA, or the combination of both hormones. While ABA antagonizes the ERF branch of the JA pathway and thus *PDF1.2* expression, it simultaneously synergizes the MYC branch of the JA pathway, typically resulting in enhanced expression of the JA‐responsive marker gene *VSP2* (Caarls, Pieterse, & Wees, 2015, Verhage et al., 2011). Figure 5b shows that in Col‐8, ABA significantly enhanced the level of MeJA‐induced *VSP2* expression, confirming previous findings (Vos, Verhage et al., 2013). However, this synergistic effect was not observed in mutant *At1g16310,* confirming that ABA–JA crosstalk is affected in this mutant. To test if this is due to an altered sensitivity to ABA, we tested the ABA responsiveness of T‐DNA insertion mutant *At1g16310* by determining the expression level of the ABA‐responsive marker gene *RAB18* (Ghelis et al., 2000) in response to exogenous application of ABA. *RAB18* mRNA accumulated to significantly lower levels in ABA‐treated *At1g16310* plants than in similarly‐treated Col‐8 plants, suggesting that reduced sensitivity to ABA may play a role in the altered ABA–JA crosstalk phenotype of *At1g16310*.

In *Arabidopsis*, the synergistic interaction of ABA on the MYC branch of the JA pathway is associated with increased resistance to herbivory (Vos et al., 2015). Feeding by the leaf‐chewing insect *M. brassicae* induces the MYC‐branch and enhances the expression of the ABA–JA responsive gene *VSP2* (Pangesti et al., 2016). To test whether the impaired ABA–JA crosstalk phenotype of mutant *At1g16310* is associated with changes in the level of resistance against *M. brassicae* feeding, we performed an insect‐resistance bioassay with this herbivore. One first‐instar *M. brassicae* caterpillar was placed on each plant and allowed to feed for 14 days, after which the caterpillar was weighed. Figure 5d shows that the caterpillars were significantly heavier when they fed from *At1g16310* plants than when they fed from Col‐8 plants. These results indicating that reduced ABA–JA crosstalk in mutant *At1g16310* is associated with enhanced susceptibility to *M. brassicae* feeding.

## 3 DISCUSSION

Plant hormones have pivotal roles in the regulation of plant defense responses. Their signalling pathways cross communicate, which provides plants with an enormous regulatory potential to rapidly adapt to their biotic and abiotic environment (Reymond & Farmer, 1998). Hormonal crosstalk is thought to be a cost‐saving strategy and may have evolved as a means of the plant to reduce allocation costs by repression of unnecessary defenses that are ineffective against the attacker that is encountered (Thaler et al., 2012; Vos et al., 2015). In *Arabidopsis,* the JA response pathway is particularly sensitive to antagonism by the plant hormones SA and ABA, which potentially impacts the level of JA‐dependent resistance against necrotrophic pathogen and herbivorous insects (Pieterse, et al., 2012, Vos, Pieterse et al., 2013). In this study, we used the level of expression of the well‐characterized JA‐responsive marker gene *PDF1.2* as a readout to mine the natural genetic variation amongst 349 *Arabidopsis* accessions for novel players in SA–JA and ABA–JA cross‐talk. The worldwide collection of natural accessions of *Arabidopsis* have a high degree of variation in plant development, physiology, and adaptation to their biotic and abiotic environment (Alonso‐Blanco et al., 2009), which makes it an ideal species to study natural variation for adaptive traits (Bergelson & Roux, 2010). Here, we show that *Arabidopsis* accessions display a large genetic variation in the magnitude by which SA and ABA affect MeJA‐induced *PDF1.2* gene expression (Figure 1d and 1e). Of the 349 accessions tested, 266 accessions displayed both SA‐ and ABA‐mediated antagonistic effects on *PDF1.2* expression (Figure 1f), which suggests that the antagonistic effects of both SA and ABA on the JA pathway must have important functions in nature. We observed only a weak correlation between the levels of SA‐mediated and ABA‐mediated antagonism on the JA pathway. Moreover, the genomic regions that our GWA mapping found to be associated with SA–JA and ABA–JA crosstalk did not overlap. Together, these findings suggest that the negative effects of SA and ABA on JA‐responsive gene expression are based on distinct molecular mechanisms and can function independently with magnitudes that depend on the genetic background.

GWA mapping is a useful tool to study the genetic architecture of traits but has not often been used for the detection of genetic variants associated with plant defense (Bartoli & Roux, 2017). Using a selection pipeline of GWA and LSM, we identified six genomic regions with significant SNP‐trait associations for SA–JA crosstalk and six genomic regions with significant SNP‐trait associations for ABA–JA crosstalk. The SA–JA crosstalk‐related SNPs were in LD with eight candidate genes, while the ABA–JA crosstalk‐related SNPs were in LD with 28 candidate genes. By testing homozygous T‐DNA insertion mutants, three of these consistently showed an altered phenotype in terms of SA‐ or ABA‐mediated suppression of MeJA‐induced *PDF1.2* expression and the level of resistance against the necrotrophic pathogen *B. cinerea* or the insect herbivore *M. brassicae*. Although the other T‐DNA insertion mutants did not show consistently different phenotypes than wildtype Col‐8, we cannot definitely conclude that they are false positives from the GWA analysis. It might be that the Col‐8 alleles of the respective genes do not have a strong effect on the SA/ABA–JA crosstalk phenotype and that the effects should be tested in the extreme accessions of the HapMap collection to reveal their effects on hormone crosstalk.

We identified the glyoxalase GLYI4 as novel player in SA–JA crosstalk. T‐DNA insertion mutant *glyI4* was insensitive to SA‐mediated suppression of MeJA‐induced *PDF1.2,* while it displayed wild‐type levels of SA‐induced *PR‐1* expression. The lack of SA‐mediated antagonism on the JA pathway in *glyI4* was associated with an enhanced level of resistance against the necrotroph *B. cinerea*. Infection of *Arabidopsis* by *B. cinerea* is typically accompanied by massive production of JA and the activation of the JA gene regulatory network, which is required for defense against this pathogen (Coolen et al., 2016; La Camera et al., 2011; Windram et al., 2012). Previously, *B. cinerea* infection was also shown to result in SA biosynthesis and signalling, possibly to exploit the SA–JA antagonism as a strategy to cause disease development (El Oirdi et al., 2011; La Camera et al., 2011). GLYI4 is a member of the Glyoxalase I enzyme family that consists of 22 members in *Arabidopsis* (Mustafiz, Singh, Pareek, Sopory, & Singla‐Pareek, 2011). Glyoxalases are involved in the detoxification of methylglyoxal, a cytotoxic ketoaldehyde that is formed as by‐product of glycolysis, lipid peroxidation, and oxidative degradation of glucose (Kaur, Ghosh, Pareek, Sopory, & Singla‐Pareek, 2014; Li, Cohenford, Dutta, & Dain, 2008; Speer et al., 2003) and accumulates during stress responses (Yadav, Singla‐Pareek, Ray, Reddy, & Sopory, 2005; Yadav et al., 2005). GLYI enzymes use glutathione to convert methylglyoxal to S‐D‐lactoylglutathione. Redox modulation, for example, via changes in glutathione levels, plays an important role in SA–JA crosstalk (Caarls et al., 2015; Koornneef et al., 2008). It is, therefore, tempting to speculate that GLYI4 modulates SA–JA crosstalk by interfering with this process.

T‐DNA insertion mutant analysis pointed to the type‐B ARR (Arabidopsis Response Regulators) ARR11 as a second novel player in SA–JA crosstalk. Mutant *arr11* displayed hypersensitivity to SA‐mediated suppression of *PDF1.2* and enhanced susceptibility to *B. cinerea* infection. The type‐B family of ARRs play a pivotal role in the early transcriptional response of plants to cytokinin (Argyros et al., 2008). Cytokinin is involved in many plant developmental processes, but it also functions as part of the hormonal network that regulates the balance between plant growth and adaptation to stress (Giron, Frago, Glevarec, Pieterse, & Dicke, 2013; O'Brien & Benkova, 2013). For instance, another member of the ARR family, the cytokinin‐activated transcription factor ARR2, has been shown to bind to the SA response factor TGA3, therewith enhancing SA/NPR1‐mediated defense gene expression and plant immunity to the biotrophic pathogen *Pseudomonas syringae* (Choi et al., 2010). Our data support a role for ARR11 as negative regulator of SA–JA crosstalk, thus positively affecting resistance against *B. cinerea*. However, the mode of action of ARR11 in this process remains elusive.

The T‐DNA insertion mutant for *At1g16310* was the only mutant with an altered phenotype related to ABA–JA crosstalk. It showed a reduced negative effect of ABA on MeJA‐induced *PDF1.2* and, reciprocally, a reduced positive effect of ABA on MeJA‐induced *VSP2*. In line with this, the level of resistance against the insect herbivore *M. brassicae* was also reduced in this mutant. Mutant *At1g16310* plants also appeared to be less sensitive to ABA, as exemplified by a reduced expression of the ABA‐responsive gene *RAB18*. In *Arabidopsis*, the synergistic interaction of ABA on the MYC branch of the JA pathway is associated with enhanced expression of *VSP2* and increased resistance to herbivory, while it antagonizes the ERF branch of the JA pathway, resulting in suppression of JA‐induced *PDF1.2* (Bodenhausen & Reymond, 2007; Pangesti et al., 2016; Vos, et al., 2015). Our data support a role for At1g16310 in enhancing the sensitivity of the plant to ABA, therewith stimulating the level of ABA–JA crosstalk and the level of resistance against insect herbivory. The protein encoded by *At1g16310* is member of the cation diffusion facilitator family of proteins, which are important for the maintenance of cation homeostasis in bacteria, yeast, plants, and mammals. The role of cation diffusion facilitators in modulating cellular cation concentrations can impact diverse processes, including cation tolerance, oxidative stress resistance, and protein turnover (Delhaize et al., 2007). However, the molecular mechanism by which At1g16310 influences ABA sensitivity and as such ABA–JA crosstalk and herbivore resistance, is yet unknown.

In this GWA study, we pinpointed several loci in the *Arabidopsis* genome that are associated with variation amongst *Arabidopsis* accessions in the antagonistic effect of SA and ABA on the JA pathway. It is tempting to speculate that the underlying genes have been subject to evolutionary selection to shape the output of the JA signalling pathway and maximize survival under the prevailing environmental conditions. Future research will be focused on unraveling the mode of action of the identified genes and their corresponding proteins in hormonal interplay, which will increase our understanding of the ingenious ways by which plants adapt to their often hostile environment.

## 4 MATERIALS AND METHODS

### 4.1 Plant material and growth conditions

In this study, a total of 349 natural *Arabidopsis thaliana* accessions of the HapMap collection (Table S1) were used, which are genotyped for ~250k bi‐allelic SNPs (Baxter et al., 2010; Chao et al., 2012; Platt et al., 2010). After quality control and imputation, this SNP‐set was reduced to a set of 214.051 SNPs (Thoen et al., 2017). Seeds of the *Arabidopsis* accessions were sown in cultivation containers filled with autoclaved river sand. Sand was supplied with half‐strength Hoagland solution containing 10 μM Sequestreen (CIBA‐Geigy, Basel, Switzerland) as described (Van Wees, Van Pelt, Bakker, & Pieterse, 2013). To attain a high relative humidity (RH) for germination, cultivation containers were enclosed in a tray with water and covered with a transparent lid. Seeds were stratified for 2 days at 4 °C in the dark to ensure a homogeneous germination after which the tray was moved to a growth chamber with an 8‐hr day/16‐hr night rhythm, a temperature of 21 °C, and a light intensity of 100 μmol m^‐2^ sec^‐1^. After 8 days, the lids of the trays were slightly opened and gradually removed over a 2‐day period to adjust to the 70% RH present in the growth chamber. Ten‐day‐old seedlings were transplanted to individual pots containing an autoclaved mixture of river sand and potting soil (1:1 [v:v]). Pots were supplied with water from the bottom up 3 times per week. At an age of 3 weeks, the plants were supplied once a week with half‐strength Hoagland solution.

### 4.2 Hormone treatment

Hormone treatments of the 349 *Arabidopsis* accessions were performed by dipping the leaves of 4‐week‐old plants in a solution containing either 0.1 mM MeJA (Serva, Brunschwig Chemie, Amsterdam, the Netherlands), or a combination of 0.1 mM MeJA and either 1 mM SA (Mallinckrodt Baker, Deventer, the Netherlands) or 0.05 mM ABA (Sigma, Steinheim, Germany), supplemented with the surfactant 0.015% (v/v) Silwet L‐77 (Van Meeuwen Chemicals BV, Weesp, the Netherlands). For each treatment, rosettes of three plants of each accession were harvested 24 hr after treatment, immediately frozen in liquid nitrogen, and individually stored at −80 °C until further analysis. Solutions with MeJA and/or ABA were diluted from a 1,000‐fold concentrated stock in 96% ethanol. Mock treatments were performed with a solution containing 0.1% (v/v) ethanol and 0.015% (v/v) Silwet L‐77.

Chemical induction treatments of T‐DNA insertion lines and Col‐8 were performed by dipping leaves of 5‐week‐old plants in an aqueous solution containing 0.015% Silwet L‐77 and 0.1 mM MeJA, or a combination of 0.1 mM MeJA and either 1 mM SA, or 0.05 mM ABA. Twenty‐four hours after treatment, the fifth leaf of three plants per treatment were individually harvested (three biological replicates per treatment), immediately frozen in liquid nitrogen and then stored at −80 °C until further analysis.

### 4.3 RNA extraction and RT‐qPCR

Total RNA was isolated as described (Oñate‐Sánchez & Vicente‐Carbajosa, 2008). DNAse treatment was performed by using DNAse I (Fermentas, St. Leon‐Rot, Germany) at the concentration of 0.5 U g^‐1^ RNA. RevertAid H minus Reverse Transcriptase (Fermentas, St. Leon‐Rot, Germany) was used to convert DNA‐free total RNA into cDNA. PCR reactions were performed in optical 384‐well plates (Applied Biosystems, Carlsbad, CA, USA) with an ABI PRISM® 7900 HT sequence detection system using SYBR® Green to monitor the synthesis of double‐stranded DNA. A standard thermal profile was used: 50 °C for 2 min, 95 °C for 10 min, 40 cycles of 95 °C for 15 s and 60 °C for 1 min. Amplicon dissociation curves were recorded after cycle 40 by heating from 60 to 95 °C with a ramp speed of 1.0 °C min^‐1^. Transcript levels were calculated relative to the *Arabidopsis* reference gene *PP2AA3* (Czechowski, Stitt, Altmann, Udvardi, & Scheible, 2005) using the 2^‐ΔΔCT^ method described previously (Livak & Schmittgen, 2001). Fold change in gene expression was calculated relative to the mock treatment in wild‐type plants. The Arabidopsis gene identifier (AGI) numbers of the studied genes are At5g44420 (*PDF1.2*), At5g24770 (*VSP2*), At2g14610 (*PR‐1*), At5g66400 (*RAB18*), and At1g13320 (*PP2AA3*). Primers are listed in Table S4.

### 4.4 GWA mapping

A collection of 349 accessions was used to investigate the genetic variation present within *Arabidopsis* (Baxter et al., 2010; Li, Huang, Bergelson, Nordborg, & Borevitz, 2010; Platt et al., 2010). Each of these accessions were genotyped versus the Col‐0 accession with ~214 k SNP markers (Kim et al., 2007). GWA mapping was performed on the RT‐qPCR expression values of *PDF1.2* in the double treatments versus the single treatment. To this end, log_2_ *PDF1.2* transcript levels in each of the three biological replicates were normalized with the constitutively expressed reference gene *PP2AA3*. Subsequently, the magnitude of the effect of SA or ABA on MeJA‐induced *PDF1.2* expression was calculated for each accession by dividing the normalized log_2_ *PDF1.2* expression values of the double treatments over that of the single treatment ([*PDF1.2* ^*MeJA+SA*^ */PDF1.2* ^*MeJA*^] or [*PDF1.2* ^*MeJA+ABA*^ */PDF1.2* ^*MeJA*^]), yielding a value <1 in the case of antagonistic effects and a value >1 in the case of synergistic effects on the level *PDF1.2* expression. Before performing the GWA analysis, the statistical Kolmogorov–Smirnov test was used to check if the data was normally distributed and to delete outliers from the dataset (Goh & Yap, 2009). Consequently, data of 327 accessions of the SA + MeJA dataset and of 322 accessions of the ABA + MeJA dataset were pursued to the GWA analysis.

GWA analysis was performed in the GWAPP web interface (http://gwapp.gmi.oeaw.ac.at/)using the AMM (Seren et al., 2012). AMM first performs a genome‐wide scan using the approximate inference proposed by Zhang et al. (2010) and Kang et al. (2010) and then updates the smallest 100 *p* values using an exact mixed model inference (Kang et al., 2008). This algorithm closely resembles the commonly used Efficient Mixed‐Model Association eXpedited (Kang et al., 2010). Additionally, GWA analysis was performed using Fast‐LMM, as described by Cao et al. (2011). FAST‐LMM captures all cofounders in the population structure simultaneously as LMM with the advantage to process larger dataset, making the analysis faster (Lippert et al., 2011). SNPs with a MAF < 5% were not considered in both models because of possibly elevated false‐discovery rates (Atwell et al., 2010). The GWAPP Geneviewer was used to zoom in on trait‐associated SNPs and reveal their position in the genome to pinpoint candidate genes within 15 kb upstream and downstream of the identified SNP. For each of the candidate genes, the annotations were retrieved from The *Arabidopsis* Information Resource10 (arabidopsis.org).

Locus‐specific association mapping of loci associated with SA + MeJA or ABA + MeJA interactions on *PDF1.2* expression, was performed using full genome sequences of 153 or 147 *Arabidopsis* accessions from the 1001 Genome project (http://signal.salk.edu/atg1001/3.0/gebrowser.php). Genome sequences surrounding the SNP of interest with a 30‐kb window were downloaded and aligned using Jalview (http://www.jalview.org/; Waterhouse, Procter, Martin, Clamp, & Barton, 2009). LSM was performed using GWA mapping phenotypic input data (Table S1). Furthermore, a MAF of >5% and a Kruskal–Wallis test was used for obtaining false discovery rate (FDR)‐corrected, SNP‐trait associations using R and the “p. adjust” function with the Bonferroni method (Benjamini & Hochberg, 1995).

### 4.5 DNA isolation and genotyping

T‐DNA insertion mutant lines were routinely genotyped using genomic DNA isolated with the sucrose method (Berendzen et al., 2005). Primers for T‐DNA insertion mutant genotyping were designed using the SIGnAL T‐DNA verification primer design tool (http://signal.salk.edu/cgi‐bin/tdnaexpress) and are listed in Table S4. DNA amplification was performed over 34 cycles in a Biorad Thermal cycler using Phire Hot Start II DNA Polymerase (Life Technologies, Bleiswijk, the Netherlands) and the following PCR conditions: denaturation at 98 °C for 5 s, annealing at 60 °C for 10 s, and elongation at 72 °C for 20 s. PCR products were separated by agarose gel electrophoresis.

### 4.6 Pathogen and insect bioassays

*Botrytis cinerea* strain B05.10 (Van Kan, Van 't Klooster, Wagemakers, Dees, & Van der Vlugt‐Bergmans, 1997) was grown for 2 weeks on half‐strength potato dextrose agar (PDA; Difco Laboratories, Leeuwarden, the Netherlands) plates containing penicillin (100 μg ml^‐1^) and streptomycin (200 μg ml^‐1^) at room temperature as described previously (Van Wees et al., 2013). *B. cinerea* spores were subsequently collected, filtered through glass wool, and resuspended in half‐strength potato dextrose broth (PDB; Difco Laboratories, Leeuwarden, the Netherlands) to a final density of 1 × 10^5^ spores ml^‐1^. After a 3‐hr incubation period, 5‐week‐old plants were inoculated by applying 5‐μl droplets of the spore suspension to six leaves of each plant. Plants were placed under a lid to increase RH to 100% to stimulate the infection. Three days after *B. cinerea* inoculation, lids were removed and the symptoms were scored in four disease severity classes ranging from no symptoms (Class I), nonspreading lesion (Class II), spreading lesion (Class III), up to severe spreading lesions with tissue maceration (Class IV) (Van Wees et al., 2013).

For caterpillar performance assays, *M. brassicae* was reared on artificial diet as described (Pangesti, Pineda, Dicke, & Loon, 2015). Growth of *M. brassicae* larvae was assessed over a period of 14 days. To this end, a single freshly‐hatched first‐instar (L1) larvae was placed on each plant. After 14 days of growth, larval weight was measured as described (Hickman et al., 2017).

## Supporting information


**Supplemental Table S1.** *PDF1.2* gene expression data in 349 *Arabidopsis thaliana* accessions treated with MeJA or a combination of MeJA and either SA or ABA.
**Supplemental Table S2.** *Arabidopsis thaliana* loci of SNP‐trait associations and underlying candidate genes within 15 kb up‐ and down‐stream of identified SNPs associated with ABA/JA crosstalk.
**Supplemental Table S3.** List of T‐DNA insertion lines used in this study.
**Supplemental Table S4.** List of primers used in this study**.**
**Supplemental Figure S1.** Relative *PDF1.2* expression in T‐DNA insertion lines of candidate genes associated with SA‐JA crosstalk.
**Supplemental Figure S2.** Relative PDF1.2 expression in T‐DNA insertion lines of candidate genes associated with ABA‐JA crosstalk.
