# Supplementary material for: Genome‐wide association study reveals novel players in defense hormone crosstalk in Arabidopsis
Source: Plant Cell Environ. 2018 Jul 3;41(10):2342–56. doi: 10.1111/pce.13357 (PMC6175328; doi:10.1111/pce.13357)
Supplement: Supplementary file 1 — Supplemental Table S1. PDF1.2 gene expression data in 349 Arabidopsis thaliana accessions treated with MeJA or a combination of MeJA and either SA or ABA. Supplemental Table S2. Arabidopsis thaliana loci of SNP‐trait associations and underlying candidate genes within 15 kb up‐ and down‐stream of identified SNPs associated with ABA/JA crosstalk. Supplemental Table S3. List of T‐DNA insertion lines used in this study. Supplemental Table S4. List of primers used in this study. Supplemental Figure S1. Relative PDF1.2 expression in T‐DNA insertion lines of candidate genes associated with SA‐JA crosstalk. Supplemental Figure S2. Relative PDF1.2 expression in T‐DNA insertion lines of candidate genes associated with ABA‐JA crosstalk. [file PCE-41-2342-s001.zip › Supplemental TableS4.docx]

Table S4. Primers used in this study

| Primer name | 5’-3’ sequence |
| --- | --- |
|  |  |
| SAIL_556_B02_LP | TTTTGCGAATTTTGTTTCCAG |
| SAIL_556_B02_RP | CAATAGAGACAGCTTGCTCGG |
| SALK_020847_LP | ATCGGTTGTTGGTCTGATGAG |
| SALK_020847_RP | TGGAAATTAGAAGAACGTGCG |
| SALK_062362_LP | TTGCAAATGGGAAGAACTTTG |
| SALK_062362_RP | AGCACCATCCAAATCACTCAC |
| SALK_121470_LP | TCATCCATTTCTCCTCTGTGC |
| SALK_121470_RP | AAAATGATCAGCGACCATGAC |
| SALK_071852_LP | TATTACCAAAACCATCGACGG |
| SALK_071852_RP | CAAGTACGATGGATCTCCCAG |
| SALK_091715_LP | GGAAGAGTGTCCGGAATTAGG |
| SALK_091715_RP | CTCATCAGTGAGCGAGTCTCC |
| SALK_075469_LP | ATTCATGATCCAGTTTGCTGC |
| SALK_075469_RP | TCTGCGTCTCGCTCTCTAAAC |
| SALK_104013_LP | CAAGTAGCCCGAGTTTTGTTG |
| SALK_104013_RP | AGCAATTGTTCCACTTTTTCG |
| SALK_052006_LP | AAATCAGAGCCATGACCAAATAC |
| SALK_052006_RP | CAAGCAACTTTGCTATCAGGG |
| SALK_106172_LP | TTAAGCCCATTAACATGCGAC |
| SALK_106172_RP | AAATACTGTCGCCCCATAACC |
| SALK_008052_LP | GAATTCCACTGCTCTGACAGC |
| SALK_008052_RP | GGTCTTTGTCGTTTCCTCCTC |
| SALK_030917_LP | CCAAAAAGTTAAACCCACTTCC |
| SALK_030917_RP | ATATTTAAGCTGGGGATTCGC |
| SALK_067593_LP | TTGGATTGTTAATCAAAGGCG |
| SALK_067593_RP | CACTTGGATCCCTCCTTCTTC |
| SALK_080726_LP | TGGGTTGTCTCTGTTAGGTGG |
| SALK_080726_RP | ATACGCATGTGGTGGGTTTAG |
| SALK_133654_LP | TCAATAGACTTCAACGTGCCAC |
| SALK_133654_RP | CTCTGTGGAGCGAGTAGTTGG |
| SALK_035774_LP | TTGTTACCCGTCTCTTGGTTG |
| SALK_035774_RP | TAGCAATGTGGTTTAGTTGCG |
| SALK_006544C _LP | ATACCAATTAAGGTGGGCTGG |
| SALK_006544C _RP | ATCATAAGTTTGTCAACGCCG |
| SALK_056712_LP | GAGTGACTTTCTTTGGGACCC |
| SALK_056712_RP | ATGGACCGCTATGATTGACTG |
| LBb1.3 | ATTTTGCCGATTTCGGAAC |
| *PDF1.2* Fw | CACCCTTATCTTCGCTGCTCTT |
| *PDF1.2* Rv | GCCGGTGCGTCGAAAG |
| *PR-1* Fw | CTCGGAGCTACGCAGAACAACT |
| *PR-1* Rv | TTCTCGCTAACCCACATGTTCA |
| *RAB18* Fw | GAGTACGGAAATCCGATGGG |
| *RAB18* Rv | CCACCTGATCCGTACCCTTG |
| *VSP2* Fw | ACGGAACAGAGAAGACCGAC |
| *VSP2* Rv | TCTTCCACAACTTCCAACGG |
| *PP2AA3* Fw | TAACGTGGCCAAAATGATGC |
| *PP2AA3* Rv | GTTCTCCACAACCGCTTGGT |
